# Proteomic Analysis of Sputum from Patients with Active Tuberculosis

**DOI:** 10.3390/proteomes13030043

**Published:** 2025-09-12

**Authors:** Endrei Marcantonio, Amy M. Woron, A. Christian Whelen, Sladjana Prisic

**Affiliations:** 1 School of Life Sciences, University of Hawaii at Mānoa, Honolulu, HI 96822, USA; 2 Hawaii Department of Health, Pearl City, HI 96782, USA

**Keywords:** tuberculosis, calprotectin, sputum

## Abstract

Background: Patients with pulmonary tuberculosis (TB) typically produce sputa, which are used to identify the pathogen. Sputum also contains host proteins that may aid in diagnosis. We hypothesized that sputa from TB patients will have unique proteomes when compared to other lung diseases. Methods: Sputa were collected from 219 patients with suspected TB. Neutrophil-derived protein calprotectin (CP), which was used as a marker for lung damage, was quantified and compared between TB and non-TB groups. Three sputa with high or low CP from each group were selected and analyzed using label-free proteomics. Results: There was no difference in CP amounts between TB and non-TB groups. However, TB samples had other differentially abundant neutrophil-associated proteins. Compared to low CP, samples with high CP had much smaller number of proteins that could differentiate between TB and non-TB groups. Only two proteins, MUC5AC and MMP8, were more abundant in TB samples, regardless of CP levels. Conclusions: Our findings suggest that TB sputa may have unique proteomes that depend on CP levels, which should be further validated due to the small sample size. Therefore, controlled and more advanced TB may need a different set of biomarkers to reliably distinguish TB from other lung diseases.

## 1. Introduction

Tuberculosis (TB) is an infectious disease in humans that is caused by *Mycobacterium tuberculosis* bacteria (Mtb), which usually affects lungs [1]. TB is clinically diagnosed as active or latent TB where patients with active TB are symptomatic (e.g., productive and often bloody sputum), while patients with latent TB are asymptomatic [2]. According to the World Health Organization, there were 10.8 million cases of active TB worldwide and 1.25 million people died due to active TB in 2023 [3]. Furthermore, antibiotic treatment remains lengthy and the sole TB vaccine, BCG, is ineffective in preventing TB in most populations [4]. Therefore, investigating TB is a pressing need to prevent further human suffering and death. Potential clues may lie in the sites of infection wherein Mtb reside.

During lung infection, Mtb is localized in granulomas, which are organized cellular structures consisting primarily of macrophages alongside various other immune cells (e.g., T cells, B cells, etc.) [5]. As the infection and disease advance, neutrophils are recruited in excess, causing necrosis of granulomas and accumulation of neutrophil debris in granulomas [5,6]. Necrotic granulomas are enriched in a neutrophil protein called calprotectin (CP) [6]. Importantly, granuloma necrosis often precipitates lung cavitation where the lung tissue is degraded and the contents of granulomas are released into the lung airways [7]. Thus, the composition of the lung environment reflects disease progression and could aid in diagnosis.

The diagnosis of active TB is based on chest X-ray and identification of Mtb from sputum, either directly by microscopy and PCR, or analysis after cultivation (e.g., DNA sequencing for identification) [3]. Typically, the sputum is treated with a mucolytic (i.e., reducing) agent and sodium hydroxide to break down and decontaminate the sputum, respectively. Following this step, the sputum is centrifuged to pellet and recover the bacteria (i.e., Mtb). Since the sputum supernatant contains proteins (and other molecules) that are not pelleted during centrifugation, they could be used for further analysis instead of discarding them. The proteomic composition of the supernatant could reflect the lung environment and therefore have insights into the stages of TB disease progression. In this study, it was hypothesized that the proteomes of sputum supernatants in TB patients will be sufficiently different to be used to discriminate pulmonary TB versus (vs.) other (non-TB) lung diseases. We also segregated patients with high CP (more inflammation/lung damage) vs. patients with low CP (less inflammation/lung damage) to dissect proteomes that depend on disease severity. We found distinct sets of differentially abundant proteins in pairwise comparisons between groups with high or low CP and in TB vs. non-TB patients that could facilitate discovery of TB biomarkers; and identified TB-specific pathways/processes that could aid in better understanding of TB pathogenesis.

## 2. Materials and Methods

Collection of sputum samples:

This study was conducted in collaboration with the Hawaii Department of Health and Diagnostics Laboratory Services, Inc. (DLS). Sputum samples were collected from suspected active TB patients from Hawaii and the U.S.-affiliated Pacific Island jurisdictions of Guam, Commonwealth of Northern Mariana Islands, American Samoa, Republic of Palau, Republic of the Marshall Islands, and/or Federated States of Micronesia. The 219 samples were de-identified (except for the results of the TB tests), and therefore this study was not considered human subject research by the University of Hawaii Institutional Review Board (protocol 217-00213) and Queen’s Medical Center. The samples were analyzed by DLS under contract with the Hawaii Department of Health, Centers for Disease Control and Prevention, or private clients to determine the smear grade (0–4+ number of acid-fast bacilli in a sputum smear as determined by microscopy), detect Mtb DNA as well as rifampin resistance by GeneXpert MTB/RIF (Cepheid, Sunnyvale, CA, USA), and culturing (Table S1). Briefly, the sputum samples were treated with NAC-PAC^®^ RED (AlphaTec, Vancouver, WA, USA). Equal volume of NAC-PAC^®^ RED reagent was added to the sputum samples, mixed for 30 s, and incubated for 15–20 min to digest and decontaminate the sample. A neutralizing buffer was added until the color of the sputa changed from red to colorless. The sputum samples were centrifuged at 3000× *g* for 15 min, the supernatants were collected and heat inactivated at 80 °C for 30 min, transported to the University of Hawaii at Mānoa, and stored at −80 °C until analysis.

Confirmation of heat inactivation:

Ten microliters of undiluted supernatant samples were inoculated on Middlebrook 7H9 (BD Difco, Franklin Lakes, MI, USA) supplemented with 10% albumin, dextrose, and catalase (ADC) enrichment (BD Difco). Agar medium was incubated at 37 °C for >3 weeks to confirm that Mtb were heat-inactivated prior to manipulation in a Biosafety Level 2 laboratory.

Quantification of calprotectin:

CP subunit S100A8 was quantified in sputum supernatant samples using a sandwich-based Enzyme-Linked Immunosorbent Assay (ELISA) that was designed and used to quantify CP in sputum supernatant samples in our previous study [8]. Sheep polyclonal anti-S100A8 (R&D Systems, Minneapolis, MN, USA) and mouse monoclonal anti-S100A8 (Santa Cruz Biotechnology, Dallas, TX, USA) were used as the capture and detection antibodies, respectively. Recombinant human CP (from Dr. Walter Chazin) was used to prepare the standards. The capture antibody was incubated in high antibody-binding 96-well white plates (Thermo Fisher Scientific, Waltham, MA, USA) for 16 h at 4 °C. The plates were washed using phosphate-buffered saline with 0.05% Tween 20 (PBST) and blocked using 3% bovine serum albumin (BSA) in PBST at 22 °C for 2 h. The CP standards and sputum samples were prepared in 3% BSA in PBST and added to the plate and incubated at 22 °C for 2 h. The plates were washed using PBST and detection antibody was added and incubated at 22 °C for 2 h. The plates were washed using PBST and incubated with goat anti-mouse IgG antibody with horseradish peroxidase (Thermo Fisher Scientific) at 22 °C for 1 h. The plates were washed to remove excess secondary antibody and Super Signal^TM^ ELISA Pico Substrate (Thermo Fisher Scientific) was added. Chemiluminescence was measured using a BioTek plate reader. The mass of CP was converted into picomoles (pmol) using its molecular mass of 24,077 Da and normalized against the total amount of protein (μg), as measured using the Bradford assay (Thermo Fisher Scientific). Note that the range of CP concentrations in TB-positive samples were published previously [8].

Preparation of proteins for mass spectrometry (MS):

A total of 12 samples were selected for proteomics analysis: “TB” and “non-TB” with high or low concentrations of CP, with 3 samples in each group. The sample size was limited to 3 due to the limited size of the cohort and the high cost of the analysis. Samples were selected based on the amount of CP relative to their cohort, i.e., samples with top 3 highest and 3 lowest CP concentrations in TB and non-TB groups were selected. Other mycobacterial infections were excluded and only samples with 0.1 mg/mL total protein were considered for proteomics. Supernatant proteins were precipitated using trichloroacetic acid with 0.1% sodium deoxycholate method [9] and resuspended in 9.5 M urea and 2% CHAPS at pH 8.5 buffer. Protein concentrations were quantified using the Bradford assay (Thermo Fisher Scientific). Each sample was processed in triplicate, resulting in 36 samples in total. The proteins were prepared for mass spectroscopy by the filter-aided sample preparation (FASP) method [10], with some modifications [11]. Proteins were reduced using a solution of 250 mM Tris(2-carboxyethyl)phosphine (TCEP) and 500 mM TEAB (ThermoFisher). TCEP-TEAB was added at a ratio of 1:5 to the protein samples (e.g., 1 µL TCEP-TEAB per 5 µL sample), and incubated at 55 °C for 1 h. Reduced samples were loaded onto Microcon-10 kDa filter units with Ultracell-10 membranes (MilliporeSigma, Burlington, MA, USA) that were pre-wet with 9.5 M urea in 100 mM Tris-HCl buffer at pH 8.5 (urea solution). Loaded columns were treated with 100 mM iodoacetamide (Bio-Rad, Hercules, CA, USA) in urea solution for 30 min at room temperature, washed using urea solution followed by 50 mM Tris-HCl at pH 8.0, and digested using Trypsin-LysC (Promega, Madison, WI, USA) in 50 mM Tris-HCl at pH 8.0 and at 37 °C for 16 h. Peptides were eluted in two sequential steps using 50 mM Tris-HCl followed by 0.5 M NaCl, which were pooled. The pooled peptides were acidified using freshly prepared 10% formic acid (~1% final concentration). The peptides were desalted using Pierce Graphite Spin Columns (Thermo Fisher Scientific) and eluted in 0.1% formic acid in 50% acetonitrile following the manufacturer’s protocol. The peptides were quantified using the Pierce Quantitative Fluorometric Peptide Assay (Thermo Fisher Scientific). Ten micrograms of peptides per sample were dried in a speed vacuum and sent to the proteomics facility at the University of California, Davis. The peptides were analyzed by liquid chromatography–tandem MS (LC-MS/MS) on a Q Exactive™ Plus Orbitrap Mass spectrometer (Thermo Fisher Scientific).

LC-MS/MS data searches and differential abundance analysis:

The LC-MS/MS data files were converted from .RAW format to .mzXML format using MSConvert (ProteoWizard 3.0) [12]. Protein identification and label-free quantification was performed using X! Tandem (The GPM, thegpm.org; version X! Tandem Alanine (2017.2.1.4)) [13] with the following search parameters: human proteome (*Homo sapiens*), precursor mass tolerance at 20 ppm, fragment mass tolerance at 10 ppm, trypsin digestion, fixed modification of cysteine (carbamidomethylation), and variable modifications of oxidation (Met), deamidation (Asn and Gln), phosphorylation (Ser, Thr, Tyr), and acetylation (Lys). The protein spectral counts were exported as .csv files. Differential abundance analyses were performed using edgeR v4.0.16 and limma v3.58.1, as we did in a previous study [8,14]. edgeR was used to normalize the unfiltered raw data (i.e., total spectral counts) to logCPM, and limma was used to determine the common dispersion using the Quantile-adjusted conditional maximum likelihood linear modeling approach [8,14]. The following groups were compared: TB vs. non-TB, TB with low CP vs. non-TB with low CP, TB with high CP vs. non-TB with high CP, non-TB with high CP vs. non-TB with low CP, and TB with high CP vs. TB with low CP. Proteins with log2 fold changes > 1 and <−1 and adjusted *p*-values (Hochberg false discovery rates) < 0.05 were called differentially abundant.

Pathway enrichment analyses:

The differentially abundant proteins were used to do pathway enrichment analyses. The KEGG and GO enrichment analyses were performed using DAVID 2021 (https://david.ncifcrf.gov/) [15,16]. Ensembl protein accession identifiers were used to conduct the searches [17]. GOplot was used to visualize the results [18].

## 3. Results

### 3.1. Proteomics of TB and Non-TB Sputa

**Figure 1:**
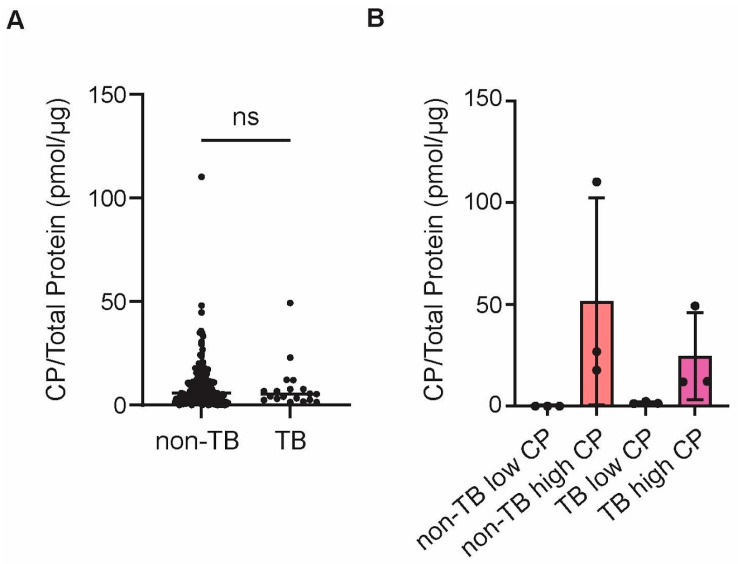
Quantification of calprotectin in sputum supernatant samples. (**A**) Concentration of CP in all TB and non-TB sputum samples. (**B**) The amount of CP in TB and non-TB sputum samples selected for proteomics analysis. Each data point is an individual sample. Samples were excluded if they were smear-tested only, or if both calprotectin and total protein were undetectable by ELISAs and Bradford assays, respectively. A Mann–Whitney test was performed to compare the conditions in panel A. ns: not significant. CP: calprotectin.

We wanted to test if CP could be used to differentiate between TB vs. non-TB sputum samples. Therefore, ELISAs were performed to quantify CP in samples in a blinded manner. TB status and diagnostic results were released after the ELISAs were performed to determine if CP was more abundant in TB vs. non-TB sputa (Table S1). Out of the 219 samples, 19 samples were TB-positive and were labeled as “TB”. Four samples were “unknown” because no further testing was performed beyond the smear test, as it was not ordered by a clinician. The rest of the samples (196) were not found to contain mycobacteria, or they were shown to have mycobacteria other than Mtb and were therefore labeled as “non-TB”. Eight samples from non-TB group had total protein and CP concentrations below the range of detection and were excluded from further analysis. When comparing TB and non-TB samples, it was found that there was no association between TB status and CP abundance (Figure 1A). In conclusion, sputum CP could not be used as a biomarker for active pulmonary TB.

To determine if there were potential biomarkers in sputum that were unique to TB, proteomics was performed on six TB sputum samples and six non-TB sputum samples, and each sample was analyzed in technical triplicates. Given that the proteome of sputum could reflect the level of inflammation and tissue destruction, three TB samples with low CP and three TB samples with high CP were selected, as shown in Figure 1B. It was assumed the amount of CP in the sputum served as a marker for the degree of neutrophilic inflammation in the lungs, i.e., active TB disease progression, as it is in blood plasma [19]. Proteomics was also performed on non-TB sputa with low and high CP to serve as controls.

The sputum proteins from TB sputa and non-TB sputa were identified and quantified using shotgun proteomics. Across all 36 samples, the average spectral counts were 14,548 ± 1962 per sample. The average spectral counts per group are listed in Table 1. Some of the most abundant proteins across all samples were albumin, hemoglobin, lactotransferrin, and immunoglobulins (Table S2).

**Figure 2:**
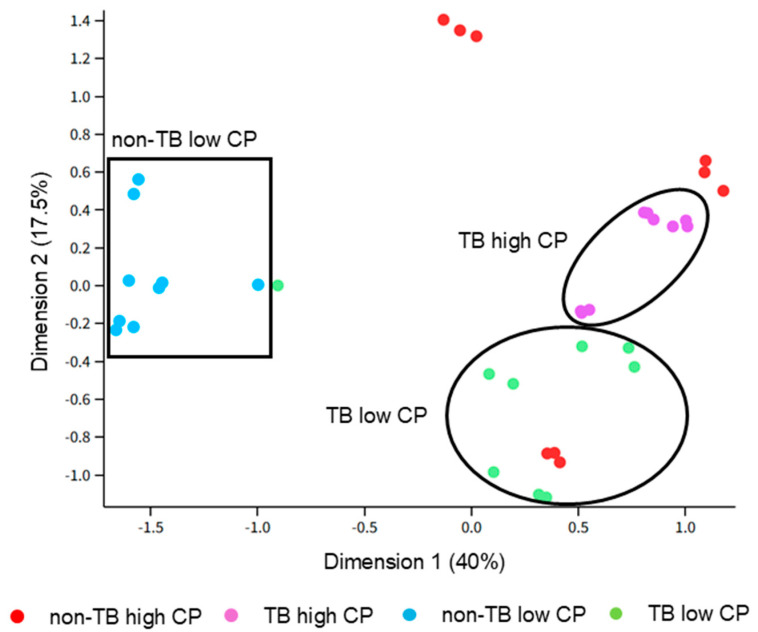
The multidimensional scaling (MDS) plot of the sputum proteomes. The dimensions correspond to the variance of the leading fold changes in the proteins across all samples. CP: calprotectin.

Differential abundance analysis was conducted, and a multidimensional scaling (MDS) plot was constructed to visualize the similarities between samples and groups. The “TB high CP”, “TB low CP”, and “non-TB low CP” groups clustered independently, while there was substantial variability of the “non-TB high CP” group (Figure 2, shown in red). The clustering pattern suggests that, except for the “non-TB high-CP” samples, each group has similarities in their proteome compositions and that they can be distinguished from other groups. All technical triplicates (i.e., processed from same sputum samples) were clustered as expected, except for one outlier of the “TB low CP” group that was more similar to the “non-TB low CP” replicates. Considering that this result suggests a technical error may have occurred (e.g., during preparation of peptides for LC-MS/MS) and there were sufficient numbers of samples needed for analysis, this sample (TB_LOW_CP_Sample3_1, see Table S2) was removed from downstream analyses.

### 3.2. Differential Abundance Analysis of TB Sputa Compared to Non-TB Sputa, Regardless of CP

The proteomes of TB and non-TB sputa, regardless of CP, were compared, and there were 64 differentially abundant proteins (DAPs), with the majority (50) upregulated (Table S3). There was upregulation of matrix metallopeptidase 8 (MMP8), grancalcin (GCA), lipocalin-2 (LCN2), myeloperoxidase (MPO), and cathepsin G (CTSG) (Table S3), which are associated with neutrophils [20]. In contrast, the strongest downregulated proteins were blood-associated proteins (e.g., carbonic anhydrase, hemoglobin subunits), which suggests that blood contamination could have been decreased in TB vs. non-TB sputa (Table S3). In summary, sputa from active TB patients had different proteomic signatures compared to sputa from non-TB patients.

**Figure 3:**
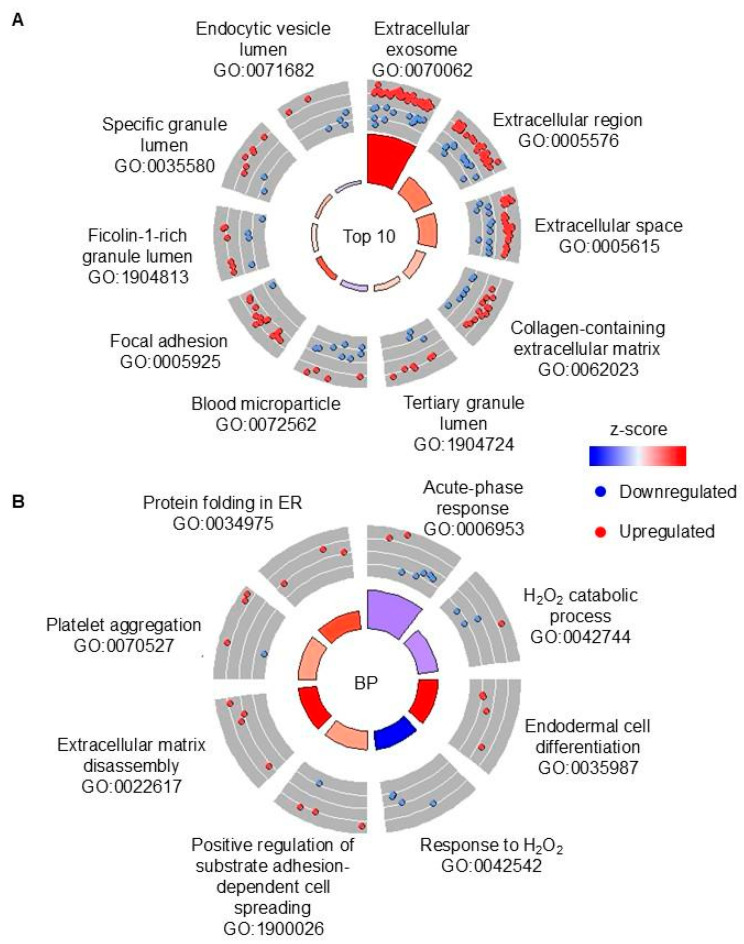
GO enrichment analysis of the DAPs in TB vs. non-TB sputa, regardless of CP. (**A**) Top 10 overall GO terms that were enriched with DAPs. All terms are cellular component terms. (**B**) GO biological process terms. All terms were sorted by lowest FDR. The bars in the circle plots depict the z-score by color and FDR by height (taller bars have lower FDRs). DAPs: differentially abundant proteins. CP: calprotectin. FDR: false discovery rate. BP: biological process. ER: endoplasmic reticulum.

In order to determine pathways that were modulated in TB vs. non-TB sputa, Gene Ontology (GO) and Kyoto Encyclopedia of Genes and Genomes (KEGG) pathway enrichment analysis were performed. DAPs were enriched in 48 GO terms (Table S4). The top 10 GO terms with the lowest false discovery rates (FDRs) are shown in Figure 3A. There was enrichment of DAPs in terms associated with phagocytosis and neutrophils (e.g., “endocytic vesicle lumen”, “ficolin-1-rich granule lumen”) (Figure 3A) [21]. There was predicted inhibition of the “blood microparticle”, suggesting there may have been less blood in the TB sputa vs. non-TB sputa. The biological process GO terms were analyzed to determine modulated host responses. DAPs were enriched in eight biological process GO terms and included “hydrogen peroxide catabolic process” and “acute-phase response” (Figure 3B). DAPs were significantly enriched in one KEGG pathway: “regulation of actin cytoskeleton” (Table S5). In summary, proteins from TB sputa identified pathways that are modified compared to non-TB sputa, including macrophage and neutrophil-associated processes.

### 3.3. Differential Abundance Analysis of TB vs. Non-TB Sputa with Low CP

**Figure 4:**
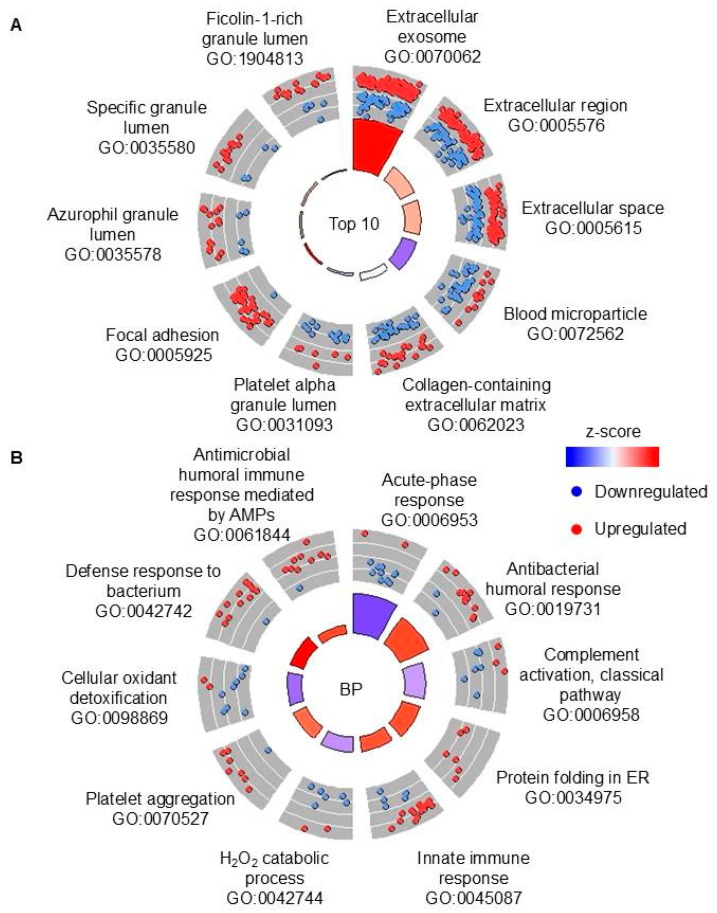
GO enrichment analysis of DAPs in TB sputa vs. non-TB sputa with low CP. (**A**) The top 10 overall GO terms that were enriched with DAPs. All terms are cellular component terms. (**B**) The top 10 GO biological process terms. All terms were sorted by the lowest FDR. The bars in the circle plots depict the z-score by color and FDR by height (taller bars have lower FDRs). DAPs: differentially abundant proteins. CP: calprotectin. FDR: false discovery rate. ER: endoplasmic reticulum. AMPs: antimicrobial peptides.

The proteomes of TB sputa and non-TB sputa with low concentrations of CP were compared to determine proteomic signatures that are present with low neutrophil-triggered inflammation. There was a much larger number of DAPs identified in this comparison than when CP was not considered: there were 181 DAPs, with the majority (127) upregulated (Table S6). Fifty-eight out of 64 DAPs out of 64 DAPs that were identified when all TB vs. non-TB samples were compared were also identified in the low CP groups (e.g., MMP8, GCA, LCN2, MPO, and CTSG). However, there was a large number (123) of DAPs that were unique for this comparison, e.g., metabolic enzymes transglutaminase 3 (TGM3), pyruvate kinase M1/2 (PKM), and glucose-6-phosphate dehydrogenase (G6PD) (Table S6). GO and KEGG enrichment analyses were performed to determine the modulated terms and pathways in TB sputa with low CP. DAPs were enriched in 170 GO terms (Table S7). In the top 10 overall GO terms, there was considerable overlap with the TB vs. non-TB (regardless of CP) comparison with all the GO terms, e.g., terms associated with blood and neutrophils (Figure 4A). There was predicted inhibition of terms associated with redox homeostasis in the top 10 biological process GO terms (Figure 4B).

KEGG pathway analysis was also performed to determine modulated pathways. There were 22 KEGG pathways that were enriched with DAPs (Table S8). The top 10 KEGG pathways with the lowest false discovery rates (FDRs) were visualized (Figure S1). There was predicted inhibition of the complement and coagulation cascades, as well as predicted activation of carbon metabolism (e.g., glycolysis) (Figure S1). In summary, there might have been differences in redox homeostasis, adaptive immunity, and carbon metabolism in TB sputum compared to non-TB sputum with low CP.

### 3.4. Differential Abundance Analysis of TB vs. Non-TB Sputa with High CP

We sought to determine if there were potential biomarkers and modulated pathways in TB sputa compared to non-TB sputa when CP was abundant (i.e., when neutrophilic inflammation was high). There was a total of 10 DAPs, with the majority (9) upregulated (Table 2). Pathway enrichment analyses were not performed due to the low number of DAPs. Therefore, the comparison of TB vs. non-TB sputa with high CP was inconclusive. However, 8 out of 10 DAPs were found in TB vs. non-TB comparisons, either in low CP (Table S6) or when all samples were compared regardless of CP (Table S3). There were only two proteins, mucin 5AC (MUC5AC) and matrix metallopeptidase 8 (MMP8), that were common in all three comparisons.

### 3.5. Differential Abundance Analysis of Non-TB Sputa with High CP vs. Non-TB Sputa with Low CP

Before comparing TB sputa with high CP vs. TB sputa with low CP, the proteomes of non-TB sputa with high CP vs. non-TB sputa with low CP were compared to determine differentially abundant proteins and modulated proteins that would be due to the degree of neutrophilic inflammation. There were 164 DAPs, with the slight majority (88) more abundant in high vs. low CP in non-TB groups (Table S9). Some of the upregulated DAPs were neutrophil-derived proteins such as MPO, CTSG, and, as expected, CP subunits S100A8 and S100A9 (Table S9). There was also downregulation of multiple immunoglobulins (e.g., IGKV1-5, IGHG3). GO and KEGG enrichment analyses were performed to determine modulated pathways. DAPs were enriched in 131 GO terms (Table S10). In the top 10 overall GO terms, DAPs were enriched in GO terms associated with granules (Figure S2A). In the top 10 biological process GO terms, there was predicted inhibition of immunoglobulin production and oxidative stress responses (Figure S2B). Regarding the KEGG pathway analysis, DAPs were enriched in 25 pathways (Table S11). The KEGG pathway analysis showed that DAPs were enriched in KEGG pathways associated with “complement and coagulation cascades” and “carbon metabolism”, which were predicted to be inhibited and activated, respectively (Figure S3). In summary, there might have been differences in numerous processes, such as redox homeostasis, adaptive and innate immunity, and carbon metabolism in non-TB sputum with high CP vs. non-TB sputum with low CP.

### 3.6. Differential Abundance Analysis of TB Sputa with High CP vs. TB Sputa with Low CP

It was hypothesized that the concentration of CP in sputum could serve as a predictor of neutrophilic inflammation in the lungs, which is associated with disease progression [22,23]. Therefore, it was postulated there would be modulated proteins and pathways in TB sputa with high CP compared to TB sputa with low CP that would be associated with neutrophils and with other processes that could serve as predictors of lung damage. Proteomes of TB with high CP vs. TB with low CP sputa were compared to determine DAPs and modulated pathways that could serve as biomarkers of disease progression. There was a total of 83 DAPs, with 40 upregulated DAPs and 43 downregulated DAPs (Table S12). Calprotectin subunit S100A9, MMP8, and MMP9 were upregulated in TB sputa with high CP, while cathepsin D was downregulated (Table S12). Several immunoglobulins were also differentially abundant, with many downregulated in TB with high CP (Table S12). Pathway enrichment analyses were performed using GO and KEGG enrichment analyses. DAPs were enriched in 53 GO terms (Table S13). In the top 10 overall GO terms, DAPs were enriched in terms related to neutrophil granules (e.g., tertiary granule lumen), which were not predicted to be activated nor inhibited (Figure S4A, Table S13). There were five GO BP terms that were associated with adaptive immunity, the immune response, platelets, actin cytoskeleton, and leukocyte adhesion (Figure S4B). DAPs were enriched in six KEGG pathways, including host responses to the infection (Figure S5 and Table S14).

Furthermore, we examined the DAPS that were unique to TB with high CP vs. TB with low CP by eliminating DAPs that were shared with non-TB with high CP vs. non-TB with low CP (Table S15). Out of the 83 DAPs, 33 were unique to TB with high CP (Table S15). The GO and KEGG analyses were performed using unique DAPs. It was found that DAPs were enriched in GO terms associated with granule lumens and exosomes (Table S16). The unique DAPs were enriched in one KEGG term “salivary secretion” (Table S17). Therefore, there might have been differences in the immune responses as revealed by proteins in TB sputum with high CP compared to TB sputum with low CP.

## 4. Discussion

The purpose of this study was to compare proteomes from sputum samples from patients with active pulmonary TB and from those with other lung diseases. We also investigated if CP could be used as a biomarker for active TB, when compared to other lung diseases, and if the proteomes of TB vs. non-TB sputa could be delineated when CP was high or low. The ultimate goal was to determine if TB sputa have different amounts of CP and other proteins that can serve as biomarkers for active pulmonary TB.

Over 200 sputa were collected from suspected TB patients, which were later shown to contain less than 10% of TB-positive samples. CP was quantified in all samples and there was no statistically significant difference between TB and non-TB samples regarding the abundance of CP. However, no additional information was available for these patients and, therefore, it is not clear what could have caused elevated CP in non-TB samples or if TB patients were already treated with antibiotics, or not, at the time of sputum collection, which could have affected CP levels as well [24]. Considering that CP is a general marker of inflammation [25] and all patients were suspects for TB, likely because they had lung conditions that triggered sputum formation and other typical symptoms of lung inflammation, it is not surprising that CP cannot be used as a specific marker for TB. However, future studies could determine if sputum CP can be used as a biomarker for successful treatment of pulmonary TB.

There were four groups of samples that were selected for further analysis using proteomics: (1) TB with high CP, (2) TB with low CP, (3) non-TB with high CP, and (4) non-TB with low CP. We first compared TB vs. non-TB samples, regardless of the CP amount, in order to identify potential biomarkers of pulmonary TB that would distinguish it from other lung diseases. Although blood in sputum is one of the typical symptoms of TB, blood-associated proteins were less abundant in TB samples, when compared to non-TB samples, which was also confirmed with pathway analyses. The lung conditions that non-TB patients had were not disclosed, so it is likely that they had symptoms severe enough to cause bloody sputum. In contrast, several proteins that are associated with neutrophils were shown to be more abundant (e.g., MPO and LCN2), as well as neutrophil-associated processes being over-represented in the pathway analyses, although CP, which is the most abundant neutrophil-derived protein, was not more abundant in TB vs. non-TB samples, as stated above. Similarly, the results from this study suggest that matrix metalloproteinases (i.e., MMP8 and MMP9) are upregulated in TB sputum vs. non-TB sputum, which is in agreement with previous reported studies that implicated these enzymes in active pulmonary TB (reviewed in [26]). Therefore, although markers of neutrophil-driven inflammation may not be specific for TB, certain neutrophil-derived proteins may be useful biomarkers.

There is a small number of published studies describing sputum proteomics in TB and they show a limited overlap with each other [27]. Using sputum samples from 10 TB patients from Ethiopia to compare their proteomes to latently infected or community controls, the authors identified 50 DAPs [28]. Only one protein, MMP8, was also implicated as a potential biomarker of active TB (Table S18). The follow-up study by the same group with a larger cohort identified 103 DAPs in pulmonary TB vs. latent TB infection, with 9 (including again MMP8) matching our findings and noting the neutrophil-driven inflammation, as we did in our study [29] (Table S18). Another previously reported proteomics analysis of sputum samples from India identified 25 DAPs, 5 of which are also found in our study, although the authors did not report if they were increased or decreased [30]. Surprisingly, this study reported decreased MMP9 in active TB [30] (Table S18). Next, sputum and salivary samples from 9 TB patients from Spain were compared to latent and uninfected controls to show DAPs in pairwise comparisons between these three groups [31]. There were 21 and 19 DAPs that overlapped with our findings in active TB vs. latent TB or TB vs. uninfected control group, respectively [31] (Table S18). These include proteins associated with neutrophils such as MMP8, GCA, and LCN2 [20,32]. Interestingly, MMP9 was increased in TB vs. latent TB, but decreased in TB vs. uninfected [31]. Since, in our study, it was not disclosed if individuals from non-TB group were latently infected (e.g., if they were PPD-tested), we cannot tell if this was the reason for this difference in comparisons. Regardless, some, but not all, neutrophil-derived proteins, such as MMP8, could be more universal biomarkers for pulmonary TB.

Next, we were interested in dissecting the host response using CP levels as a proxy for disease severity caused by neutrophil accumulation. There were more DAPs when comparing samples with low CP (181), with only 10 DAPs when comparing TB vs. non-TB samples with high concentration of CP. Six out of these 10 DAPs in high-CP samples are also DAPs in low-CP samples, including MMP8, but not MMP9, which was not elevated in TB vs. non-TB sputum samples with high CP. If all three comparisons are considered, i.e., regardless of CP, low CP or high CP, mucin 5AC is the only other protein (in addition to MMP8) that is significantly more abundant in TB vs. non-TB. Therefore, these two proteins (MMP8 and MUC5AC) may be insensitive to disease severity. However, MUC5AC is found to be less abundant in TB sputa in several other studies [29,31], while MMP8 is consistently shown to be more abundant in active TB across multiple studies [28,29,31]. The discrepancy with MUC5AC may have been due to differences in the patient populations. This present study analyzed samples collected from the Pacific Basin, whereas the other studies used samples from Ethiopia and Spain [29,31]. In addition, we only had samples from six patients in each group. Hence, the increased abundance of MUC5AC could have been due to population diversity and/or small sample size. It does not seem probable that our sample decontamination protocol specifically contributed to this discrepancy as well. However, it is possible that our sample treatment affected the overall results compared to other studies because we subjected our samples to strong alkaline treatment, whereas other studies did not [29,31]. In general, efforts to improve reproducibility between sputum proteomic studies should be made. Sample collection is usually performed by clinical providers (as was performed in this study and introduces some variability) and also prioritizes pathogen isolation over preservation of host proteins. Current proteomics analysis requires specialized expertise and equipment and is therefore complex and costly. Accordingly, sputum processing should be standardized to allow analysis of host proteins and proteomics protocols should be designed to be more affordable and accessible to researchers in low-income countries where TB burden is the highest.

Finally, we wanted to compare TB samples with high vs. low CP. There were 83 DAPs in this comparison, but 48 out of these DAPs are also found in non-TB high vs. low CP comparisons, so they are likely caused by higher inflammation in high-CP samples. Thirty-five proteins that are uniquely DAPs in high vs. low CP TB samples (including LTF that is a DAP in the opposite direction in the non-TB comparison) may provide potential biomarkers for disease severity or treatment success. Since other studies did not compare samples with high or low CP, as we did, we looked for studies in which proteomics analysis was performed during treatment, which would presumably decrease neutrophil inflammation. In one such study from South Africa, 266 DAPs were identified when proteins from bronchoalveolar lavage (BAL) from TB patients with active disease and clinically cured individuals [33]. Surprisingly, both CP subunits (S100A8 and S100A9) were not DAPs in this comparison, and 7 DAPs that are found in our study, i.e., in high vs. low CP in TB sputum samples and in BAL from patients with active TB vs. cured, are showing the opposite trend. This study was not an appropriate match for our study possibly due to different sample types being used (i.e., sputum vs. BAL) and CP amounts in BAL not being significantly different before and after treatment. The differences between our study and other studies likely reflect the highly heterogeneous nature of the disease, the study populations, sample preparation and analysis, and various other factors that influence human biology. In addition, the high heterogeneity of our non-TB cohort affected our data. To decrease the variability, future studies should use non-TB samples from patients who are not sick or stratify the non-TB samples according to the type of illness (e.g., pneumonia, chronic obstructive pulmonary disease, influenza, etc.). Overall, our study must be validated in other population cohorts due to our small sample size.

If this study were to be repeated, several changes must be made to improve the experimental design. First, the sample size should be increased to account for the intrinsic biological variability of human samples and to increase the statistical power of the analyses. Second, the samples had relatively low spectral counts (~15,000 counts per sample), and much higher numbers can be achieved with newer instruments. The low sample complexity may have contributed to the relatively small number of identified proteins, as some of the most abundant proteins were blood-associated proteins (e.g., hemoglobin subunits, albumin, immunoglobulins, etc.). This could be improved by their removal to improve the detection of proteins with lower abundances, which can be achieved using commercially available kits (e.g., Pierce™ Albumin Serum Depletion Kits from Thermo Fisher Scientific). We also used 10 kDa filters to prepare samples, which might have contributed to the low sample complexity because the low-molecular-weight proteins were not captured by the filter for digestion. Alternatively, using targeted proteomics would be more appropriate as a follow-up study, in which case low complexity will be less problematic. Third, it was initially assumed that samples with high CP had increased neutrophilic inflammation and were from patients with more advanced disease progression compared to samples with lower concentrations of CP. While neutrophils are strongly associated with TB disease progression [22,23], and serum CP is as well [19], the patient information was unknown in this study (i.e., disease progression was unknown). The lack of patient information also prevented us from choosing appropriate non-TB controls, which may be the reason for the high variability in “non-TB high-CP” group, as seen in the MDS plot. Therefore, it is possible that other patient characteristics complicated the comparison, especially when “non-TB high-CP” group was used as a control group. Thus, patient information should be known in future studies in order to minimize confounding variables and select better matched groups. Other characteristics of the patient cohort should be known as well (e.g., age, gender, HIV status, illness of non-TB patients). Lastly, even though we included standard posttranslational modifications in our analysis, we did not attempt to differentiate proteoforms and focused on ORF products only. The lack of proteoform differentiation is problematic because this possibly contributed to the low complexity of our search results and, more importantly, different proteoforms for the same genes can have different functions [34,35]. Therefore, our functional analyses remain incomplete without proteoform differentiation, which should be addressed by future studies. In summary, with an increased sample size, depletion of common proteins, and more information about the sample cohort, there would be an increased chance to identify proteins that could serve as biomarkers for TB and TB disease progression. However, MMP8 remains the most promising TB biomarker, as shown previously and in this study to be elevated in TB patients.

## Figures and Tables

**Table 1:** Overview of the proteins identified and spectral counts across the four groups. CP: calprotectin.

Group	Proteins Identified	Average Spectral Counts	Standard Deviation (±)	Coefficient of Variation
Non-TB low CP	819	11,994	869	0.07
Non-TB high CP	1853	16,000	1275	0.08
TB low CP	1551	14,186	1357	0.1
TB high CP	1865	16,011	516	0.03

**Table 2:** Differentially abundant proteins in TB vs. non-TB sputa with high CP. The two proteins noted in bold are common in all three comparisons. FC: fold change.

Gene Symbol	Ensembl ID	log2 FC	AveExpr	t	*p*-Value	Adjusted *p*-Value
**MUC5AC**	ENSP00000485659	3.47	10.92065	4.248534	0.000148	0.020954
PRR4	ENSP00000228811	3.03	11.8752	4.287908	0.000132	0.020954
IGLV3-19	ENSP00000374844	2.44	8.24343	3.87908	0.000435	0.030792
ANXA4	ENSP00000377833	2.11	7.468757	3.936153	0.000369	0.030792
**MMP8**	ENSP00000236826	1.80	8.955381	3.798247	0.000549	0.031067
OLFM4	ENSP00000219022	2.22	8.600148	3.608172	0.000941	0.043143
TLN1	ENSP00000316029	1.73	8.762223	3.515591	0.00122	0.043143
KRT1	ENSP00000252244	−1.78	10.61901	−3.52942	0.001173	0.043143
HMGB2	ENSP00000296503	1.85	8.563864	3.470662	0.001382	0.043449
CYRIB	ENSP00000429150	1.62	7.648167	3.410076	0.001634	0.046231

## Data Availability

The original data presented in this study are openly available in ProteomeXchange via the PRIDE database (http://www.ebi.ac.uk/pride) using the accession PXD065067.

## Supplementary Materials

The following supporting information can be downloaded at: https://www.mdpi.com/article/10.3390/proteomes13030043/s1. Figure S1: The top 10 KEGG pathways that were enriched with DAPs in TB vs. non-TB sputa with low CP; Figure S2: GO enrichment analysis of DAPs in non-TB sputa with high CP vs. non-TB sputa with low CP; Figure S3: The top 10 KEGG pathways that were enriched with DAPs in non-TB sputa with high CP vs. non-TB sputa with low CP; Figure S4: GO enrichment analysis of DAPs in TB sputa with high CP vs. TB sputa with low CP; Figure S5: KEGG pathways that were enriched with DAPs in TB sputa with high CP vs. TB sputa with low CP; Table S1: The smear grade, GeneXpert results, culture identification, and the amount of CP and total protein per sample; Table S2: Complete search results of sputum supernatant samples; Table S3: Differentially abundant proteins in TB vs. non-TB sputa, regardless of CP; Table S4: Gene Ontology enrichment analysis of DAPs in TB vs. non-TB sputa, regardless of CP; Table S5: KEGG pathway enrichment analysis of DAPs in TB vs. non-TB sputa, regardless of CP; Table S6: Differentially abundant proteins in TB vs. non-TB sputa with low CP; Table S7: Gene Ontology enrichment analysis of DAPs in TB vs. non-TB sputa with low CP; Table S8: KEGG pathway enrichment analysis of DAPs in TB vs. non-TB sputa with low CP; Table S9: Differentially abundant proteins in non-TB sputa with high CP vs. non-TB sputa with low CP; Table S10: Gene Ontology enrichment analysis of DAPs in non-TB sputa with high CP vs. non-TB sputa with low CP; Table S11: KEGG pathway enrichment analysis of DAPs in non-TB sputa with high CP vs. non-TB sputa with low CP; Table S12: Differentially abundant proteins in TB sputa with high CP vs. TB sputa with low CP; Table S13: Gene Ontology enrichment analysis of DAPs in TB sputa with high CP vs. TB sputa with low CP; Table S14: KEGG pathway enrichment analysis of DAPs in TB sputa with high CP vs. TB sputa with low CP; Table S15: TB-specific differentially abundant proteins in TB sputa with high CP vs. TB sputa with low CP; Table S16: Gene Ontology enrichment analysis of TB-specific DAPs in TB sputa with high CP vs. TB sputa with low CP; Table S17: KEGG pathway enrichment analysis of TB-specific DAPs in TB sputa with high CP vs. TB sputa with low CP; Table S18: Differentially abundant proteins that overlap with other studies.
