# Supplementary material for: Proteomic Analysis of Sputum from Patients with Active Tuberculosis
Source: Proteomes. 2025 Sep 12;13(3):43. doi: 10.3390/proteomes13030043 (PMC12452556; doi:10.3390/proteomes13030043)
Supplement: Supplementary file 1 [file proteomes-13-00043-s001.zip › proteomes-3797846-SI figures.pdf]

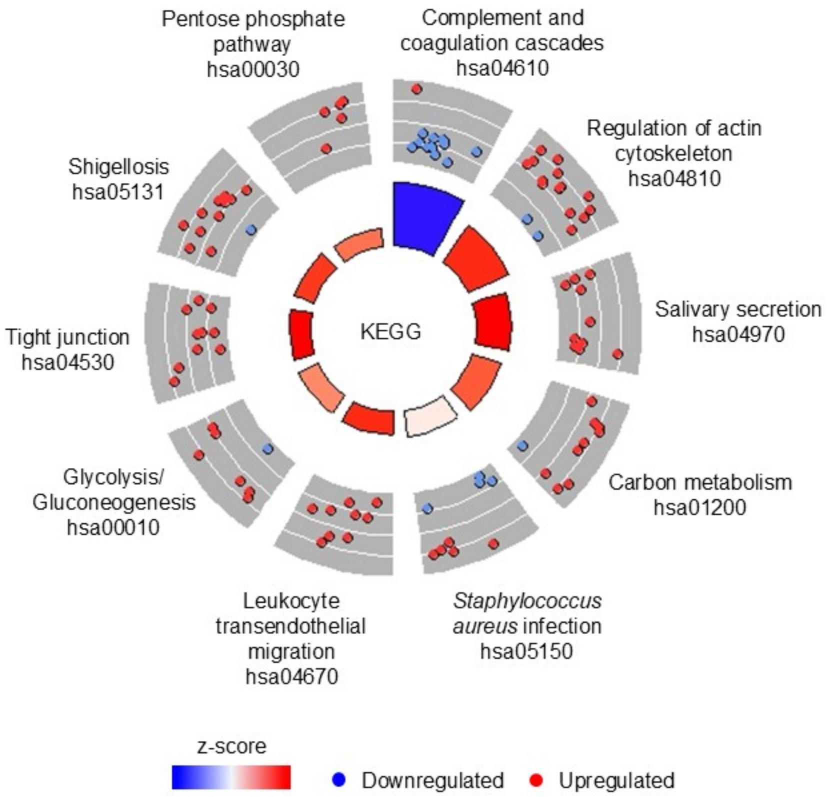

Figure S1. The top 10 KEGG pathways that were enriched with DAPs in TB vs. non-TB sputa with low CP. Top 10 pathways according to the lowest FDRs. The bars in the circle plots depict the z-score by color and false discovery rate by height (taller bars have lower false discovery rates). CP: calprotectin. FDR: false discovery rate.

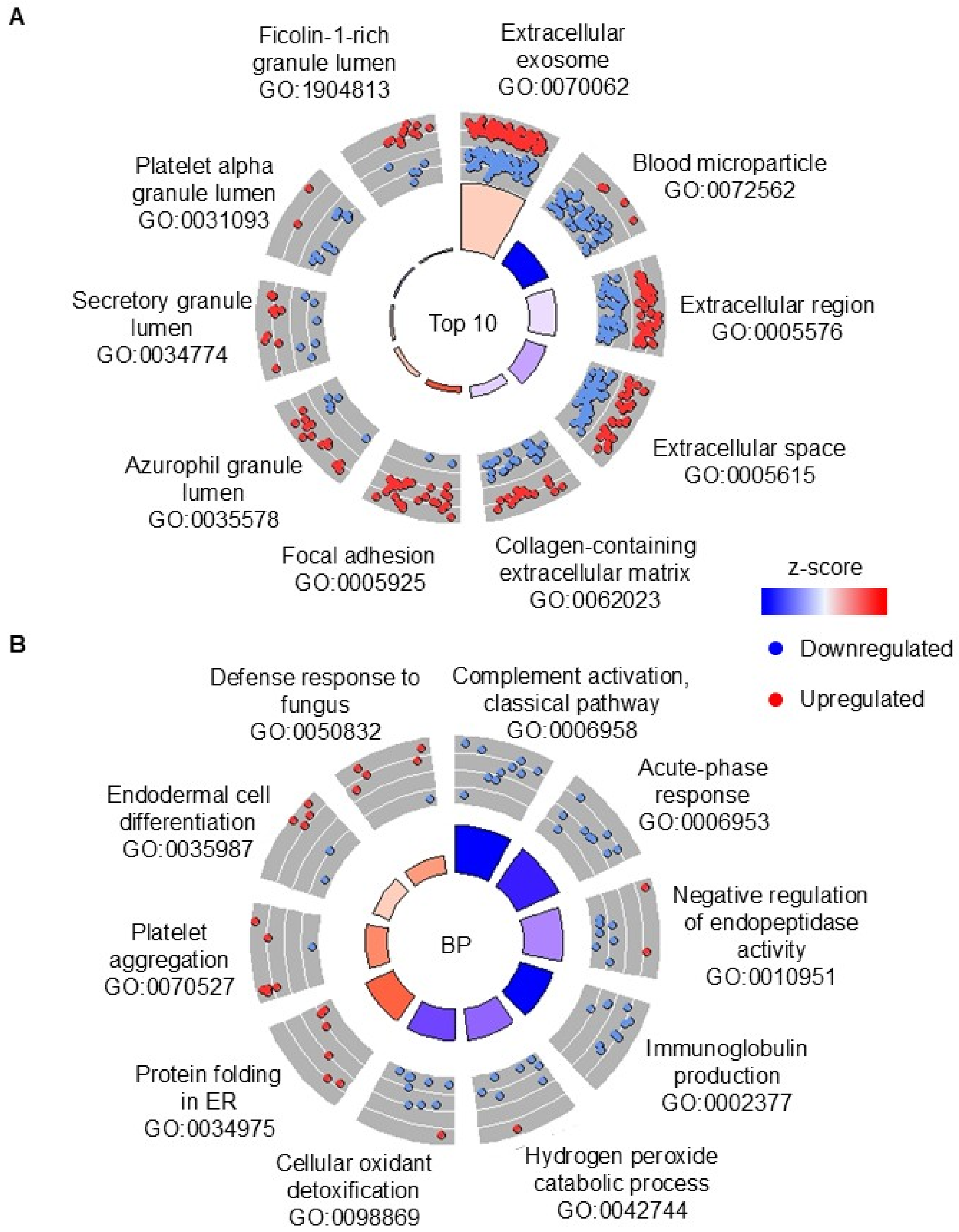

Figure S2. GO enrichment analysis of DAPs in non-TB sputa with high CP vs. non-TB sputa with low CP. A) The top 10 overall GO terms that were enriched with DAPs, sorted by the lowest FDRs. B) The eight GO biological process terms, sorted by the lowest FDRs. The bars in the circle plots depict the z-score by color and FDR by height (taller bars have lower FDRs). DAPs: differentially abundant proteins. CP: calprotectin. FDR: false discovery rate. BP: biological process.

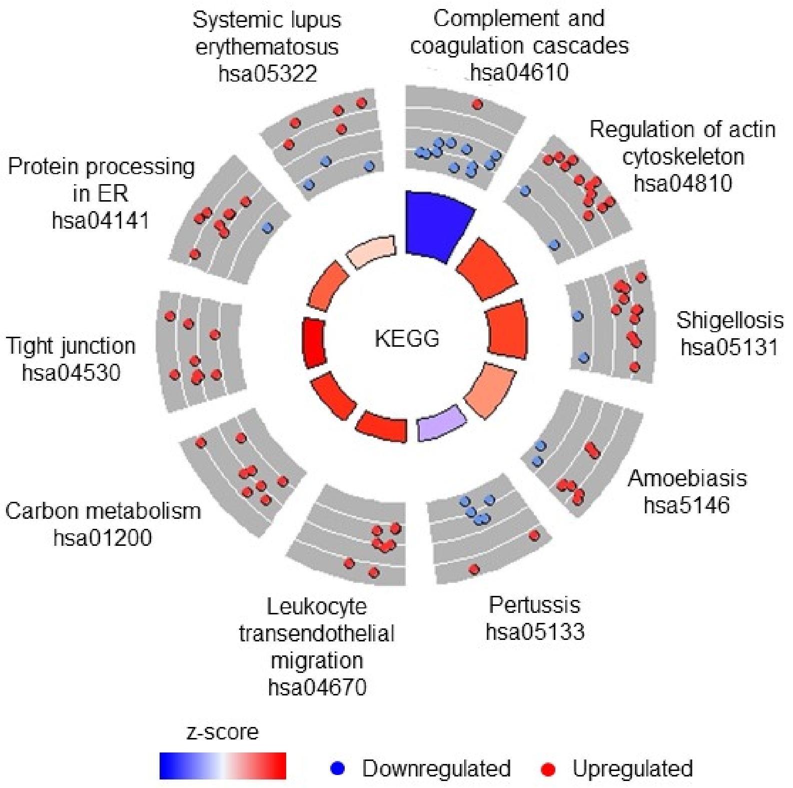

Figure S3. The top 10 KEGG pathways that were enriched with DAPs in non-TB sputa with high CP vs. non-TB sputa with low CP. Top 10 pathways according to the lowest FDRs. The bars in the circle plots depict the z-score by color and FDR by height (taller bars have lower false discovery rates). CP: calprotectin. ER: endoplasmic reticulum. FDR: false discovery rate.

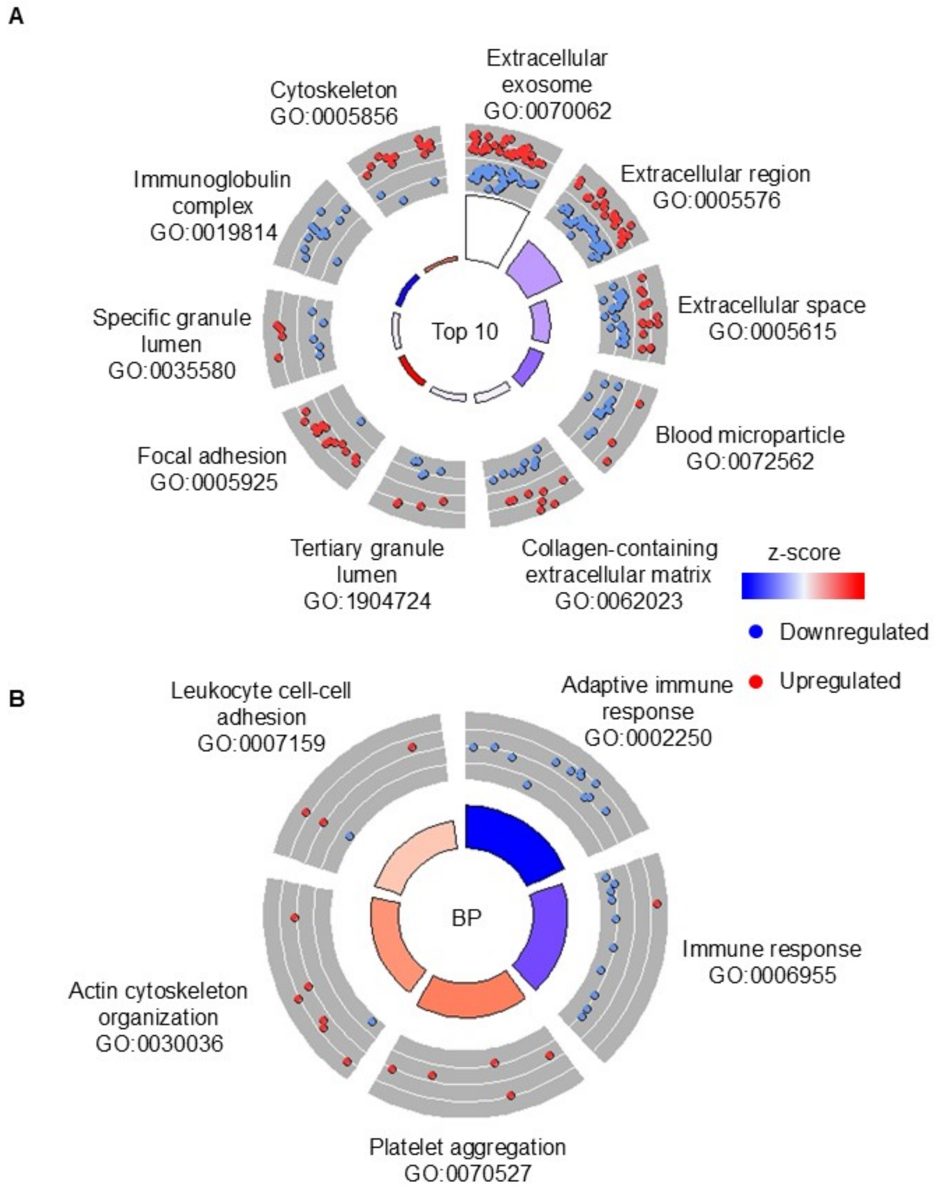

Figure S4. GO enrichment analysis of DAPs in TB sputa with high CP vs. TB sputa with low CP. A) The top 10 overall GO terms that were enriched with DAPs, sorted by the lowest FDRs. B) The five GO biological process terms, sorted by the lowest FDRs. The bars in the circle plots depict the z-score by color and FDR by height (taller bars have lower FDRs). DAPs: differentially abundant proteins. CP: calprotectin. FDR: false discovery rate.

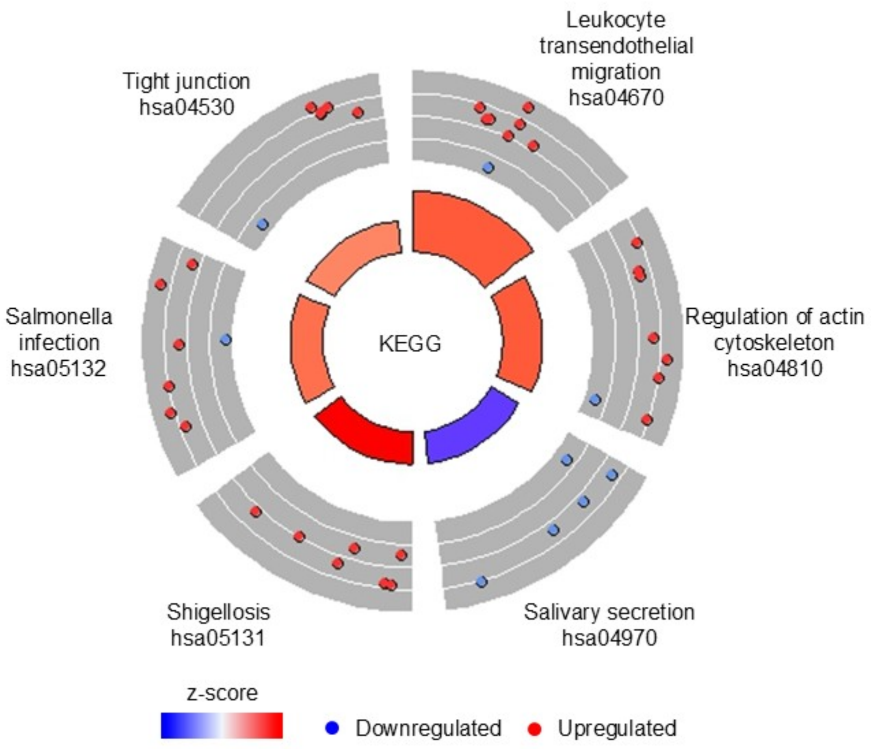

Figure S5. KEGG pathways that were enriched with DAPs in TB sputa with high CP vs. TB sputa with low CP. The pathways are sorted from lowest to highest FDR. The bars in the circle plots depict the z-score by color and FDR by height (taller bars have lower FDRs). FDR: false discovery rate.
